# Whole-exome sequencing identifies homozygous mutation in *TTI2* in a child with primary microcephaly: a case report

**DOI:** 10.1186/s12883-020-01643-1

**Published:** 2020-02-15

**Authors:** Vincent Picher-Martel, Yvan Labrie, Serge Rivest, Baiba Lace, Nicolas Chrestian

**Affiliations:** 1 Department of psychiatry and neurosciences, Centre de recherche Cervo Brain Research Centre and CHU de Québec, Laval University, 2601 chemin de la canardière, Québec, Qc G1J 2G3 Canada; 2 Centre de recherche du CHU de Québec-Universtié Laval, Québec, Qc Canada; 3 Centre de recherche CHU de Québec- Universtié Laval, Québec, Qc Canada; 4 Department of Clinical Genetic, CHU de Québec- Université Laval, Québec, Qc Canada; 5 Department of Paediatric Neurology, Paediatric Neuromuscular Disorder, Centre Mère Enfant Soleil, Laval University, Québec, Qc Canada

**Keywords:** TTI2, Microcephaly, Whole-exome sequencing, Case report

## Abstract

**Background:**

Primary microcephaly is defined as reduced occipital-frontal circumference noticeable before 36 weeks of gestation. Large amount of insults might lead to microcephaly including infections, hypoxia and genetic mutations. More than 16 genes are described in autosomal recessive primary microcephaly. However, the cause of microcephaly remains unclear in many cases after extensive investigations and genetic screening.

**Case presentation:**

Here, we described the case of a boy with primary microcephaly who presented to a neurology clinic with short stature, global development delay, dyskinetic movement, strabismus and dysmorphic features. We performed microcephaly investigations and genetic panels. Then, we performed whole-exome sequencing to identify any genetic cause. Microcephaly investigations and genetic panels were negative, but we found a new D317V homozygous mutation in TELOE-2 interacting protein 2 (*TTI2*) gene by whole-exome sequencing. TTI2 is implicated in DNA damage response and mutation in that gene was previously described in mental retardation, autosomal recessive 39.

**Conclusions:**

We described the first French Canadian case with primary microcephaly and global developmental delay secondary to a new D317V homozygous mutation in *TTI2* gene. Our report also highlights the importance of TTI2 protein in brain development.

## Background

Microcephaly is generally defined as a significant reduction in occipital-frontal head circumference (OFC). Primary microcephaly could be detectable before 36 weeks of gestation whereas secondary microcephaly is developed after birth. Primary microcephaly could be caused by infections (CMV, toxoplasma, rubella, herpes, HIV), ischemia or hypoxia, exposure to alcohol or drugs, mitochondrial mutations or autosomal recessive mutations [1]. Autosomal recessive primary microcephaly (MCPH; Microcephaly Primary Hereditary) is a rare disorder with incidence around 1 to 8/250000 live births [2]. More than 16 genes have been reported. The cause of microcephaly remains unclear in many cases after extensive investigations.

**Fig. 1:**
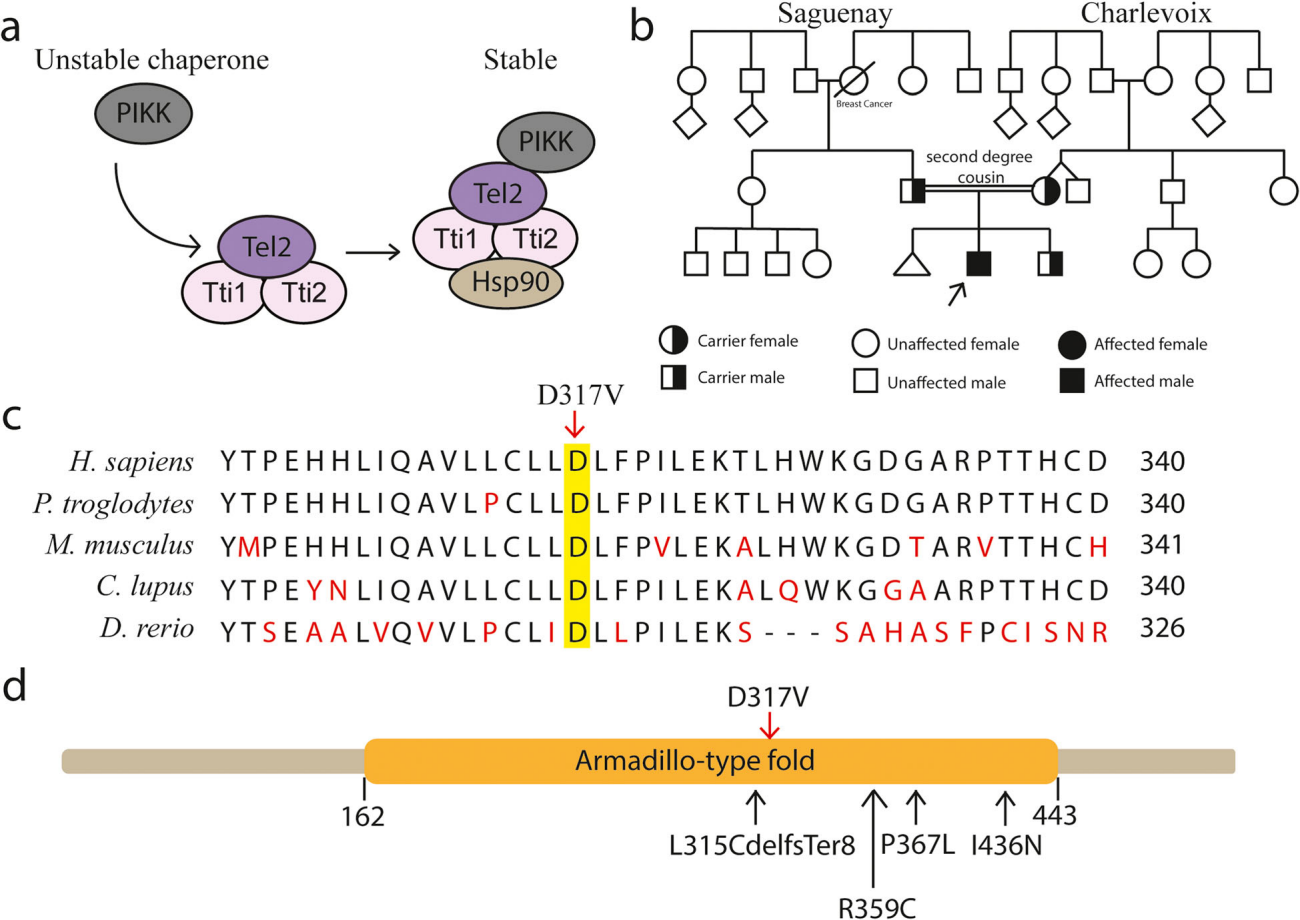
Mutation in TELO2-interacting protein 2 (*TTI2*) in patient with microcephaly. **a** Schematic representation of the cellular role of TTI2 in PIKK stability. PIKK phosphatidylinositol 3-kinase-related kinases, Tel2 TELO2, TTI1 TELO2-interacting protein 1, TTI2 TELO2-interacting protein 2, Hsp90 heat-shock protein 90. **b** Pedigree of the family from the region of Charlevoix-Saguenay in Quebec, Canada. The black arrow indicates the case presented here. For simplicity, diamonds represent more than one individual of both genders. The double line represents second degree cousin parents. **c** Evolutionary conservation in different species of TTI2 amino acid sequence in the region of D317V mutation. Comparison of *H. sapiens* (human) *TTI2* and its orthologues in *P. troglodytes* (chimpanzee), *M. musculus* (mouse), *C. lupus* (dog) and *D. rerio* (zebrafish). Non-conserved amino acids are denoted in red. The sequences are illustrated from N-terminal (left) to C-terminal (right). **d** Predicted domain of TTI2 protein. TTI2 contain only the armadillo-type fold domain. Red arrow illustrates the mutation described in this case and black arrow illustrates other mutations previously described (Table 1)

Here, we report a child with primary microcephaly carrying D317V Homozygous mutation in TELO2-interacting protein 2 (*TTI2*) gene, identified by whole-exome sequencing (WES). We described the features associated with the mutation and discussed about *TTI2* implication in neurodevelopment. TTI2 protein is implicated in DNA damage response (DDR) and is a part of the TTT complex with telomere length regulation protein TELO2 (Tel2) and TTI1. The TTT chaperone complex interact with Hsp90 to promote stability of the phosphatidylinositol 3-kinase-related kinases (PIKKs) [3–5] implicated in numerous cell functions (Fig. 1a) [10]. A mutation in *TTI2* was previously descr

ibed in mental retardation, autosomal recessive 39 in three affected siblings with microcephaly at 30–36 years of age [6].. Recently, two publications reported cases of compound heterozygous mutations, implying that the clinical spectrum of *TTI2* is evolving [8, 9]. This disorder is characterized by reduced intellectual functioning associated with impairment in adaptative behavior, delayed psychomotor development and short stature. Our patient highlights the importance of TTI2 protein in normal brain development and increases the phenotypic description of *TTI2* related microcephaly.

## Case presentation

**Fig. 2:**
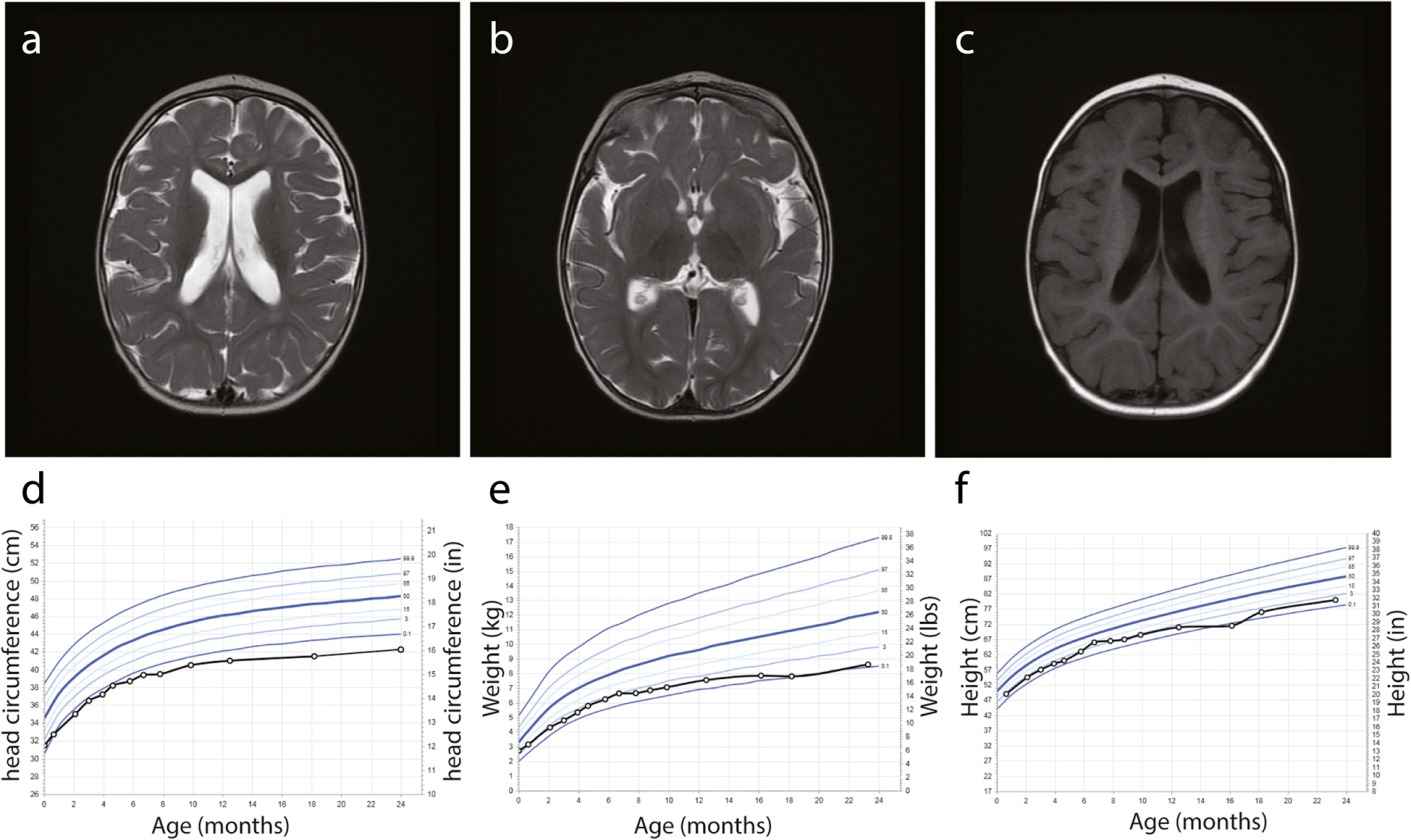
Clinical phenotype of the patient with *TTI2* mutation. **a-b** axial T2 images and **c** axial Flair image of brain magnetic resonance imaging (MRI) from the boy at 1 year of age. Small supratentorial ventriculomegaly and minor mixed diffuse cortical atrophy can be observed. **d-f** Growth curves for head circumference (**d**), weight (**e**) and height (**f**) from birth to the age of two years old. Blue line indicate percentile from age and sex matched controls

**Fig. 3:**
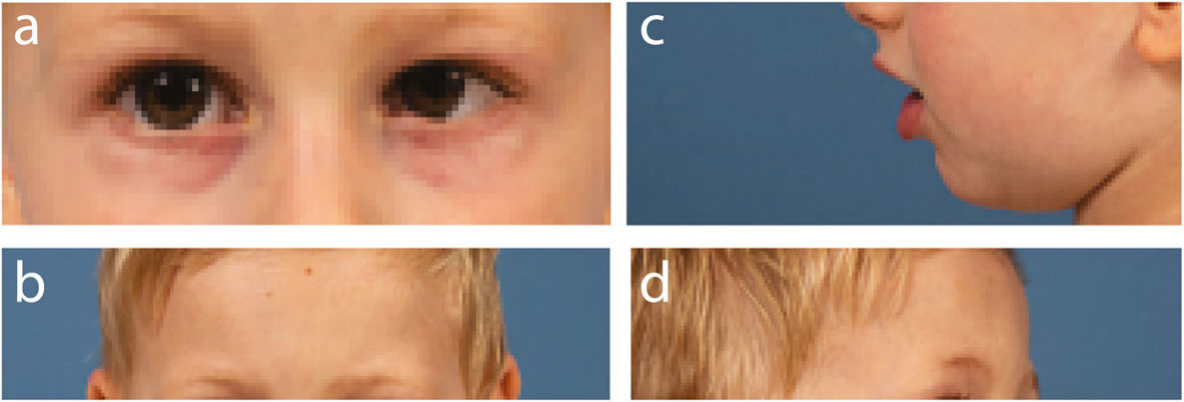
Patient’s phenotypic features. **a-d** Samples pictures of the described patient 1 month before his fourth birthday. We can denote the strabismus (**a**), a deep set of eyes (**b**), a high palate (**c**) and the forehead with prominent metopic ridge (**d**)

The patient was originally referred to our neuropediatric clinic at 11 months of age for global development delay and convergent strabismus. The boy was born from healthy second cousin French Canadian parents (Fig. 1b). The pregnancy and delivery were unremarkable except for suspected in utero microcephaly during a prenatal ultrasound at 34 weeks of gestation (<4th percentile). The boy weight 2732 kg (5th percentile) with 31,5 cm of OFC (<3rd percentile) and height of 49 cm (25th percentile) at birth (Fig. 2d-f). At 4 months of age, the parents observed hypotonia, abnormal limb choreiform and athetoid movements and strabismus. At medical evaluation, the child had language retardation with only warbles, global motor retardation, slight axial hypotonia, dyskinesia, mannerism, convergent strabismus, microcephaly with OFC at 40 cm (> 2 SD, <2nd percentile) as well as normal strength. At 1 year of age, brain imaging by magnetic resonance imaging (MRI) revealed no gross abnormality except mild supratentorial ventriculomegaly and mild to moderate mixed diffuse cortical atrophy (Fig. 2a-c). Electroencephalographic studies revealed no epileptic abnormality with normal background rhythm. At 2 years of age, the child had severe language dysfunction, severe microcephaly with OFC at 42.5 cm (< 0.1 percentile) and short stature with heighted 78.5 cm (0.7 percentile) and weighted 9.3 kg (0.1 percentile) (Fig. 2d-f). The patient also exhibits dysmorphic features including fair complexion, narrow and triangular face, forehead with prominent metopic ridge, a deep set of eyes, a high palate and a supernumerary nipple (Fig. 3). At 4 years of age, he was able to follow 2 step simple commands but without any words. He was able to recognize some letters and numbers. His gross motor development was with normal range (walking independently, climbing stairs, jumping, using tricycle) and had mild fine motor skills difficulties. He still presents short stature 81 cm (< 0.1 percentile), low weight 12 kg (< 0.1 percentile) and severe microcephaly at 43 cm (< 0.1 percentile) (Fig. 2).

Large investigations for causes of primary microcephaly were performed. No infectious causes were detected, and the lactate levels were 1.1 to 2.8 mmol/L. The investigations revealed no abnormalities in levels of urinary organic acid, in levels of blood amino acids, in acylcarnitine profile, purines and pyrimidines and sterols profile. Genetic panels were performed before whole-exome sequencing. The nuclear mitochondrial genes panel (Fulgent, Temple City, USA) included 505 genes and revealed variants in five genes. The panel revealed an heterozygous mutation in *CYP2781* which is known to be pathogenic when homozygous. The panel also revealed heterozygous variants of unknown significance in *CFTR*, *RYR*1, *AKAP10* and *SARDH*. Detection of Autosomal Recessive Primary Microcephaly Series (ASPM) (University of Chicago, USA) was negative and the autosomal recessive primary microcephaly Tier 2 sequencing and deletion/duplication panel (University of Chicago, USA) revealed autosomal recessive heterozygous variant of unknown significance in *CASC5*. The Prader-Willi and Angelman’s syndrome methylation study performed in our centre was negative (Centre Hospitalier Université Laval, Canada). Finally, no mutation was found in *MECP2* for Rett’s syndrome (University of Montreal, CHU de Ste-Justine, Canada).

Whole-exome sequencing was performed at the CRCHU de Québec- UL genomic center (Quebec, Canada) as described in materials and methods (Additional file 1). The WES identified homozygous variant in the *TTI2* gene: c.950A > T (p.Asp317Val) (Fig. 1c). The variant was exonic missense. Both parents were heterozygous for this variant and the sibling was an unaffected heterozygous carrier. The D317V homozygous variant in *TTI2* was confirmed by independent single gene sequencing (Fulgent, Temple City, USA). The variant was less frequent than 0.01% in Broad dataset. The aspartate is highly conserved within all mammals and non-mammalian vertebrates, which suggest a primordial role of aspartate in protein structure (Fig. 1c). Indeed, the change from aspartate to valine is radical with a physiochemical difference in Grantham’s Distance of 152 [11].

## Discussion and conclusions

In this study, by whole-exome sequencing, we identified the first French Canadian case with autosomal recessive homozygous *TTI2* mutation in a child with severe microcephaly, short stature, dyskinesia, convergent strabismus and dysmorphic features. We described the first homozygous c.950A > T (p.Asp317Val) mutation in *TTI2* gene causing primary progressive microcephaly and short stature. During the preparation of this manuscript, two other groups report compounds heterozygous mutations in children with intellectual disabilities and microcephaly (Table 1), but none were homozygous for this mutation [8, 9].. Another mutation, c.1307 T > A (p.Ile436Asn), in *TTI2* gene was previously described in three siblings born from healthy first cousin parents [6]. Contrary to our case, the siblings had normal neonatal period and developed progressive microcephaly with OCF reaching − 3/− 4 SD at 30–36 years old. Another missense *TTI2* mutation (p.Pro367Leu) was described in a large consanguineous Iranian family with intellectual disability [7]. The defined homozygous mutation in our case probably come from a strong founder effect found in the French Canadian from the region of Charlevoix-Saguenay, Quebec [12]. The phenotype is similar in all cases and the most frequent findings include primary or progressive microcephaly (80%), dysmorphic features (80%), severe cognitive impairment (70%), severe speech delay (70%), strabismus (70%), movement disorder (60%), short stature (60%) and scoliosis (50%) (Table 1).

**Table 1:** Summary of genetic variance in TTI2 gene

cDNA	Amino acid changes (Abbreviated)	Number of cases	Clinical characteristics	References
c.1307 T > A	p.Ile436Asn (I436N)	3	Normal Growth parameters, microcephaly at adult age, severe cognitive impairment, severe speech delay, short stature, dysmorphic features, vertebral anomalies	[6]
c.1100C > T	p.Pro367Leu (P367L)	2	Non-syndromic moderate intellectual disability	[7]
c.950A > T	p.Asp317Val (D317V)	1	Primary microcephaly, short stature, severe speech delay, dysmorphic features, strabismus, dyskinesia	Described here
Patient 1 compound c.1075C > T and c.950A > T and Patient 2 compound c.539 T > C and c.575 T > C	p.Arg359Cys and p.Asp317Val (R359C and D317V) p.Leu180Pro and p.Leu192Pro (L180P and L192P)	2	Intellectual disabilities, progressive microcephaly, high nasal bridge, deep-set eyes, partial ovarian failure	[8]
^a^Compound c.942_944delTCTins and c.1100C > T	p.Leu315CysfsTer8 and p.Pro367Leu (L315CdelfsTer8 and P367L)	2	Intellectual disabilities, microcephaly, growth retardation, speech disorder, movement disorders	[9]

^a^Complete mutation name c.942_944delTCTinsCTGTGCTTCCATTCCTTCCTCCTAG

Our study and the review of the literature suggested an important role for *TTI2* in brain development. It is noteworthy that all identified mutations are located in the Armadillo-type fold domain of *TTI2* (Fig. 1d). The superhelical structures of the Armadillo-type fold domain is necessary for binding to its large substrates including other members of the TTT complex [13]. TTI2 plays a key role in promoting the stability of the PIKK family. The PIKK family include DNA-PK, ATM, ATR, MTOR, SMG-1 and TRRAP. DNA-PK, ATM and ATR are implicated in cellular response to double-strand DNA break [14]. MTOR is implicated in metabolism, cell growth, autophagy and in maintenance of cytoskeleton [15], whereas SMG-1 is more implicated in surveillance of non-sense mRNA to prevent translation of truncated protein [16, 17]. TRRAP do not possess any kinase activity but is implicated in chromatin maintenance [18]. Patients with mutation in TTI2 exhibited decreased level and activity of all PIKK members by dysfunction of the TTI1- TTI2-TELO2 complex [6]. It is not clear how deficits in PIKK proteins can cause neurodevelopmental defect. However, studies in animal models of MCPH have revealed important role of DNA damage response in embryonic neurogenesis [19–21]. By example, premature neurogenesis leading to a reduction in the number of neuronal cells was caused by a ventricular neural stem cell defect in MCPH models [22–24]. Mutations in *TELO2* gene also caused PIKKs dysfunction and severe intellectual disability associated with microcephaly, visual and hearing impairments and abnormal movements [25].

There are numerous genes implicated in DNA reparation. Mutations in those genes can lead to pathology with similar landscapes. This include Nijmegen Breakage syndrome, ataxia-telangiectasia, DNA-ligase IV deficiency, seckel syndrome 1, Cernunnos-XLF and bloom syndrome. These syndromes generally have a combination of microcephaly, intellectual disability, short stature, skeletal abnormalities and facial dysmorphisms. The same features were observed in our patient, except for the presence of skeletal abnormalities. One of the PIKKs proteins, mTOR, is particularly implicated in dendritic translation which have important impact on spine morphogenesis and synaptic plasticity [26, 27]. Indeed, mTOR dysregulation could lead to cognitive deficits [28].

In conclusions, we described the first French Canadian case with primary microcephaly and global developmental delay secondary to a new D317V homozygous mutation in *TTI2* gene. This finding associated with other findings suggested an important role for the TTT complex in brain development. Our findings enlarge the phenotypic variability observed with *TTI2* mutations showing that *TTI2* related microcephaly could present with less disability than previously described. Though, *TTI2* should be included in any microcephaly panel to reach an accurate genetic diagnosis.

## Supplementary information

**Additional file 1.** Materials and methods. Methods for exome sequencing analysis including DNA extraction and cell line immortalization, library preparation, whole exome sequencing, bioinformatic analyses of exome data, variant filtering and sanger sequencing.

## Data Availability

The datasets used and/or analysed during the current study are available from the corresponding author on reasonable request. Anonymized data will be shared by request from any qualified investigator.

## Abbreviations

Hsp90: Heat-shock protein 90
MCPH: Microcephaly primary hereditary
OFC: Occipital-frontal head circumference
PIKKs: Phosphatidylinositol 3-kinase-related kinases
TTI2: TELO2-interacting protein 2
WES: Whole-exome sequencing
