# Supplementary material for: Whole-exome sequencing identifies homozygous mutation in TTI2 in a child with primary microcephaly: a case report
Source: BMC Neurol. 2020 Feb 15;20:58. doi: 10.1186/s12883-020-01643-1 (PMC7023720; doi:10.1186/s12883-020-01643-1)
Supplement: Supplementary file 1 — Additional file 1. Materials and methods. Methods for exome sequencing analysis including DNA extraction and cell line immortalization, library preparation, whole exome sequencing, bioinformatic analyses of exome data, variant filtering and sanger sequencing. [file 12883_2020_1643_MOESM1_ESM.docx]

## Materials and methods

### DNA extraction and cell line immortalization

Blood samples were used for genomic DNA extraction. When blood sampling was excluded due to circumstances, saliva samples were used instead. DNA was extracted using QIAamp DNA Blood kit (Qiagen, Toronto, ON, Canada) or ORAGENE-DNA OG-500 kit (DNA Genotek, Ottawa, ON, Canada) according to the manufacturer’s instructions. Lymphocytes (LCLs) were isolated and immortalized from 3-5 ml of blood samples using Epstein-Barr virus in 15% RPMI medium as previously described [1-3].

### Library preparation and whole exome sequencing

Libraries have been prepared from 3 μg of high-quality genomic DNA using SureSelect XT human All exon V6+UTR kit (Agilent Technologies, Santa Clara, USA). This technology is already automated and up-and-running at the CHUQC-UL Research Center. DNA was fragmented on a Covaris instrument (Covaris, Woburn, MA, USA) and adaptor-tagged to an average size of ~275-300bp. Libraries were then be subjected to exome capture. Three libraries with unique index were pooled together in equimolar ratio and sequenced at a mean coverage of 100X on an Illumina HiSeq2500 for paired-end 125 bp sequencing at both sites.

### Bioinformatic analyses of exome data and variant filtering

Data were processed using a pipeline adjusted from GATK Best Practices [4]. Raw data were demultiplexed using Illumina’s proprietary bcl2fastq to get to an open format. Then raw reads were trimmed using Trimmomatic [5] and mapped to human reference genome (hg19) using BWA [6]. Duplicated reads were flagged using Picard MarkDuplicates [7] and base score recalibration was performed using GATK BaseRecalibrator [8]. Variant call was first performed on individual samples using GATK HaplotypeCaller before performing multi-sample joint aggregation and re-annotation using GATK GenotypeGVCFs. Variants were functionnally annotated based on data from SiFT [9], CADD [10], avsnp, Kaviar, ExAC, esp6500siv, 1000genomes and Polyphen 2 [11] using Annovar [12]. Variants rarity was assessed with databases of variants frequencies in different populations from gnomAD, ExAC and 1000Genomes. The availability of exome data from family trios allowed identification of potential deleterious variants based on recessive, de novo and compound heterozygote transmission modes.

Moreover, all prediction tools of pathogenicity used in this study namely SIFT, PolyPhen-2, Mutation Taster and Provean identify this amino acid change as Damaging, while the Combined Annotation-Dependent Depletion (CADD) tool resulted in a score of 31, which represents a high damaging score given that a score higher than 20 could result in a protein defect. It should be noted that the CADD tool is a measure of variant deleteriousness based on several factors including the surrounding sequence context, gene model annotations, evolutionary constraint, epigenetic measurements and functional predictions. According to in sillico prediction tools this mutation is considered to be damaging but no functional test was performed in our laboratory.

### Sanger sequencing

Validation of causal variant was then performed using the Sanger sequencing technology. In brief, direct sequencing was performed on an ABI3731 automated sequencer using version 3.1 of the Big Dye fluorescent method according to the manufacturer's instructions (Applied Biosystems, Foster City, USA). Sequence data were analyzed using the Staden preGap4 and Gap4 programs. Primers used for targeted sequencing of the c.A950T (p.D317V: rs200045967, NM_025115) variant within the TTI2 gene were: Forward : 5’-GGTCCTGAGGAGAATGTATTG and Reverse : 5’-AGCAGCTCAAGCAACATAG.

References

1. Durocher, F., et al., *Mutation analysis and characterization of ATR sequence variants in breast cancer cases from high-risk French Canadian breast/ovarian cancer families.* BMC Cancer, 2006. **6**: p. 230.

2. Litim, N., et al., *Polymorphic variations in the FANCA gene in high-risk non-BRCA1/2 breast cancer individuals from the French Canadian population.* Mol Oncol, 2013. **7**(1): p. 85-100.

3. Desjardins, S., et al., *Genetic variants and haplotype analyses of the ZBRK1/ZNF350 gene in high-risk non BRCA1/2 French Canadian breast and ovarian cancer families.* Int J Cancer, 2008. **122**(1): p. 108-16.

4. Van der Auwera, G.A., et al., *From FastQ data to high confidence variant calls: the Genome Analysis Toolkit best practices pipeline.* Curr Protoc Bioinformatics, 2013. **43**: p. 11.10.1-33.

5. Bolger, A.M., M. Lohse, and B. Usadel, *Trimmomatic: a flexible trimmer for Illumina sequence data.* Bioinformatics, 2014. **30**(15): p. 2114-20.

6. Li, H. and R. Durbin, *Fast and accurate long-read alignment with Burrows-Wheeler transform.* Bioinformatics, 2010. **26**(5): p. 589-95.

7. Toolkit, P. *Picard Tools - By Broad Institute*. 2018; Available from: <https://broadinstitute.github.io/picard/>.

8. McKenna, A., et al., *The Genome Analysis Toolkit: a MapReduce framework for analyzing next-generation DNA sequencing data.* Genome Res, 2010. **20**(9): p. 1297-303.

9. Kumar, P., S. Henikoff, and P.C. Ng, *Predicting the effects of coding non-synonymous variants on protein function using the SIFT algorithm.* Nat Protoc, 2009. **4**(7): p. 1073-81.

10. Kircher, M., et al., *A general framework for estimating the relative pathogenicity of human genetic variants.* Nat Genet, 2014. **46**(3): p. 310-5.

11. Adzhubei, I., D.M. Jordan, and S.R. Sunyaev, *Predicting functional effect of human missense mutations using PolyPhen-2.* Curr Protoc Hum Genet, 2013. **Chapter 7**: p. Unit7.20.

12. Wang, K., M. Li, and H. Hakonarson, *ANNOVAR: functional annotation of genetic variants from high-throughput sequencing data.* Nucleic Acids Res, 2010. **38**(16): p. e164.
